# Supplementary material for: SAASI: Sampling Aware Ancestral State Inference
Source: Nat Commun. 2026 May 11;17:6307. doi: 10.1038/s41467-026-72851-5 (PMC13376925; doi:10.1038/s41467-026-72851-5)
Supplement: Supplementary file 1 — Supplementary Information [file 41467_2026_72851_MOESM1_ESM.pdf]

*Supplementary Note 1: Derivation of the main decomposition*

For clarity, we provide an explicit derivation of our central equation for the $A_{e,i}$,

$$A_{e,i}(\tau) = \Pr(Y_e(\tau) = i|\mathcal{T}, \theta) = \frac{\Pr(Y_e(\tau) = i|\mathcal{T}_e^C(\tau), \theta)\Pr(\mathcal{T}_e(\tau)|Y_e(\tau) = i, \theta)}{\sum_j \Pr(Y_e(\tau) = j|\mathcal{T}_e^C(\tau), \theta)\Pr(\mathcal{T}_e(\tau)|Y_e(\tau) = j, \theta)}. \quad (1)$$

In this derivation, we will suppress $\tau$ and $\theta$ to simplify notation. For example, we write $\Pr(\mathcal{T}) = \Pr(\mathcal{T}_e, \mathcal{T}_e^C)$, and $A_{e,i} = \Pr(Y_e = i|\mathcal{T})$. By Bayes' theorem, we have

$$\Pr(Y_e = i|\mathcal{T}) = \frac{\Pr(\mathcal{T}|Y_e = i)\Pr(Y_e = i)}{\Pr(\mathcal{T})}.$$

Since $\mathcal{T}_e$ and $\mathcal{T}_e^C$ are conditionally independent given $i$ (which means, given that edge $e$ is in state $i$ at time $\tau$), the above is

$$Pr(Y_e = i|\mathcal{T}) = \frac{\Pr(\mathcal{T}_e|Y_e = i)\Pr(\mathcal{T}_e^C|Y_e = i)\Pr(Y_e = i)}{\Pr(\mathcal{T})}. \quad (2)$$

Using Bayes' theorem again, we write

$$\Pr(\mathcal{T}_e^C|Y_e = i) = \frac{\Pr(Y_e = i|\mathcal{T}_e^C)\Pr(\mathcal{T}_e^C)}{\Pr(Y_e = i)}$$

and

$$\Pr(\mathcal{T}) = \Pr(\mathcal{T}_e, \mathcal{T}_e^C) = \Pr(\mathcal{T}_e|\mathcal{T}_e^C)\Pr(\mathcal{T}_e^C).$$

Substituting these into Eq. 2, we have

$$\Pr(Y_e = i|\mathcal{T}) = \frac{\Pr(\mathcal{T}_e|Y_e = i)\Pr(Y_e = i|\mathcal{T}_e^C)}{\Pr(\mathcal{T}_e|\mathcal{T}_e^C)}. \quad (3)$$

It remains to show that the denominator has the form given in (1) of the main text. Since the state of edge $e$ must be one and only one of the possible ancestral states, we have

$$\begin{aligned}\Pr(\mathcal{T}_e|\mathcal{T}_e^C) &= \sum_j \Pr(\mathcal{T}_e, Y_e = j|\mathcal{T}_e^C) \\ &= \sum_j \Pr(\mathcal{T}_e|\mathcal{T}_e^C, Y_e = j)\Pr(Y_e = j|\mathcal{T}_e^C) \\ &= \sum_j \Pr(\mathcal{T}_e|Y_e = j)\Pr(Y_e = j|\mathcal{T}_e^C)\end{aligned} \quad (4)$$

where the second line is a conditional probability, and the last line is due to the conditional independence of $\mathcal{T}_e$ and $\mathcal{T}_e^C$ given the state of edge $e$.

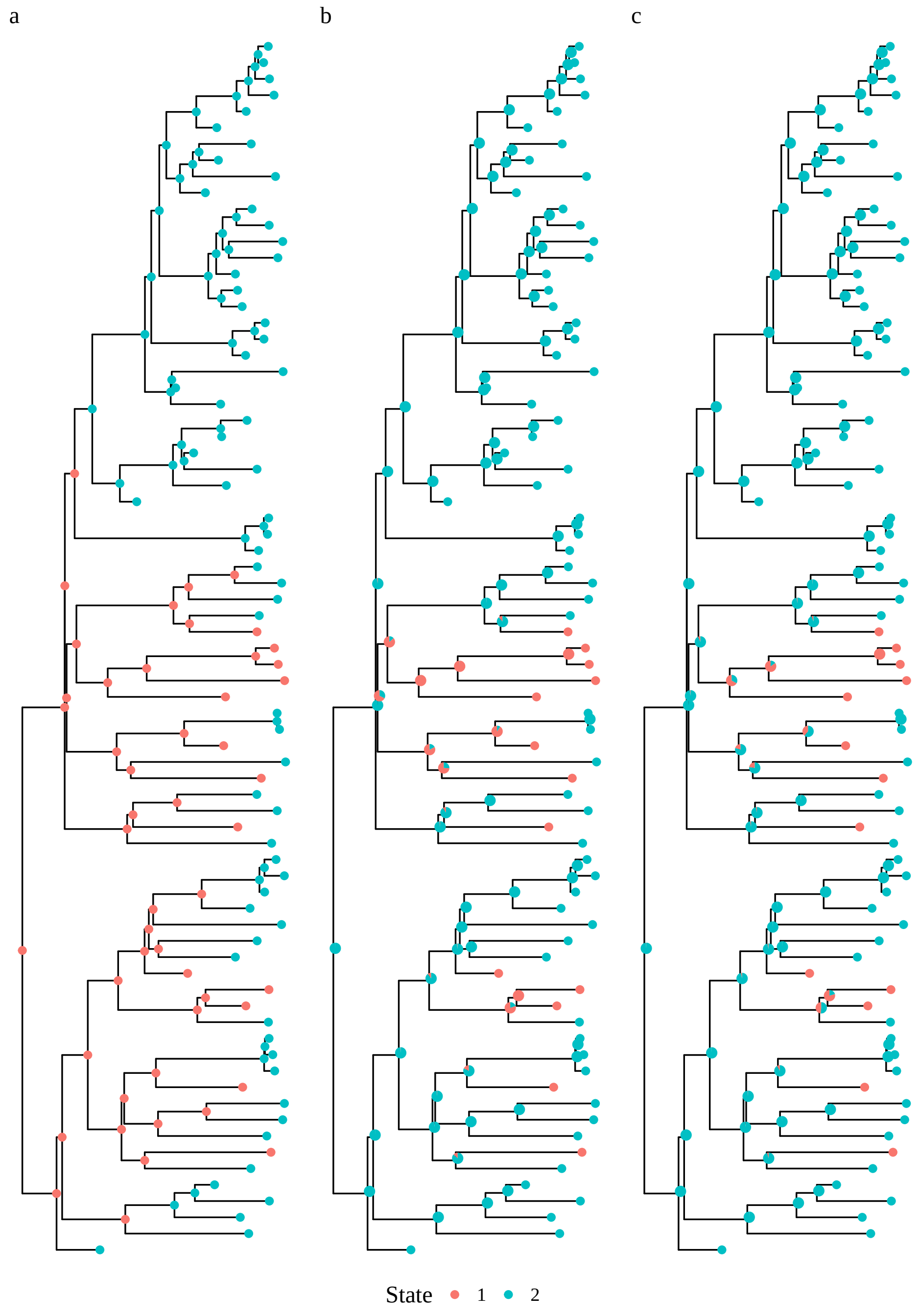


**Figure 1: Ancestral state inference using SAASI under an equal sampling model and using ace.** a: Simulated tree with known transmission histories; b: SAASI with equal sampling rates ($\psi_1 = \psi_2 = 0.5$); c: ace under equal sampling rate model. The tree is generated using the following parameters: $\lambda_1 = \lambda_2 = 1$, $\mu_1 = \mu_2 = 0.045$, $q_{12} = q_{21} = 0.05$, and $\psi_1 = 0.05$, $\psi_2 = 0.5$.

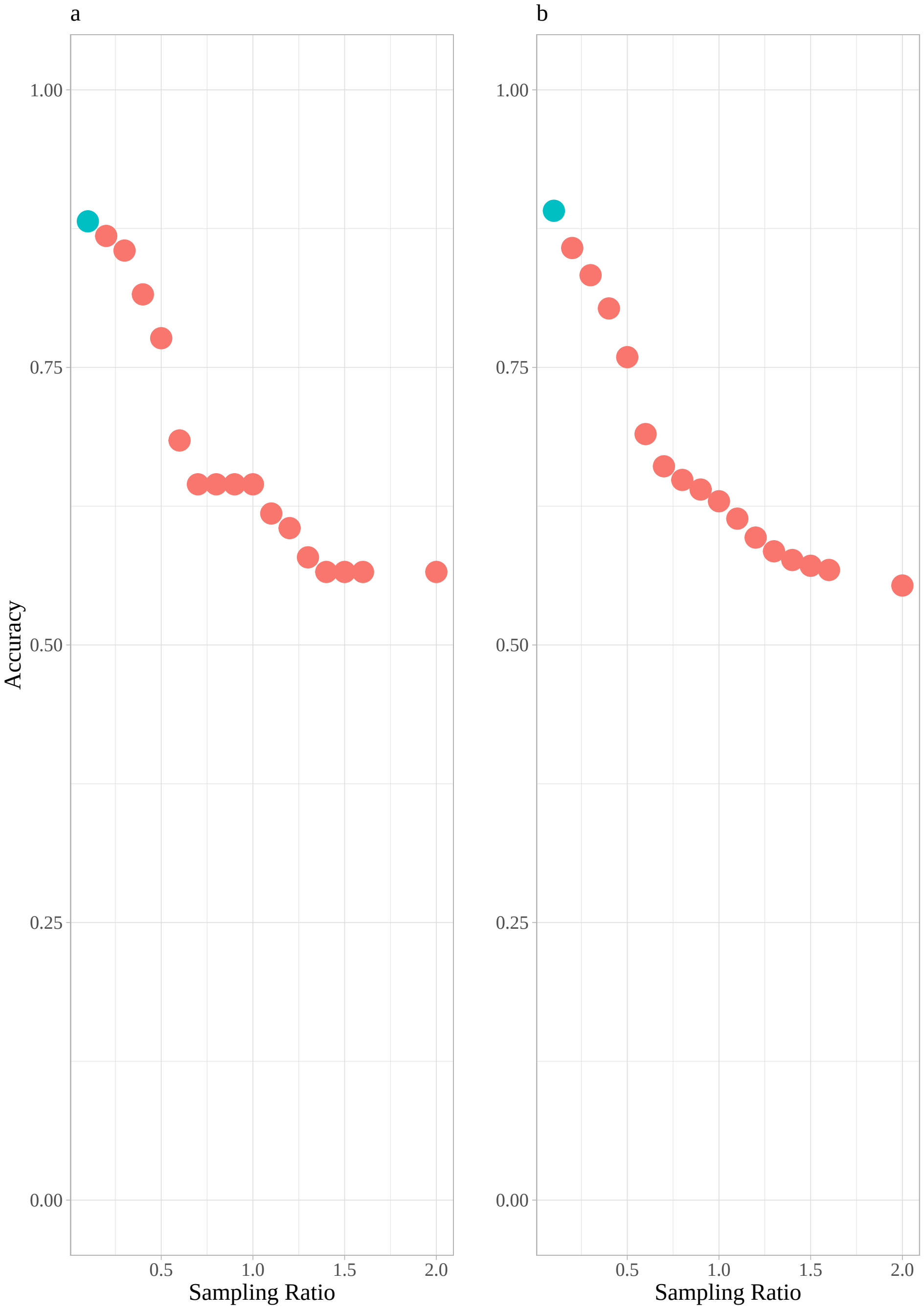


**Figure 2: Accuracies of SAASI under varying sampling ratios on a fixed simulated tree.** a: Consensus accuracy; b: Probability accuracy. The blue point represents accuracy under the true sampling ratio ($\frac{\psi_2}{\psi_1} = 0.1$). Red points represent accuracy under mis-specified sampling ratios ranging from $\frac{\psi_1}{\psi_2} = 0.15$ to 2.

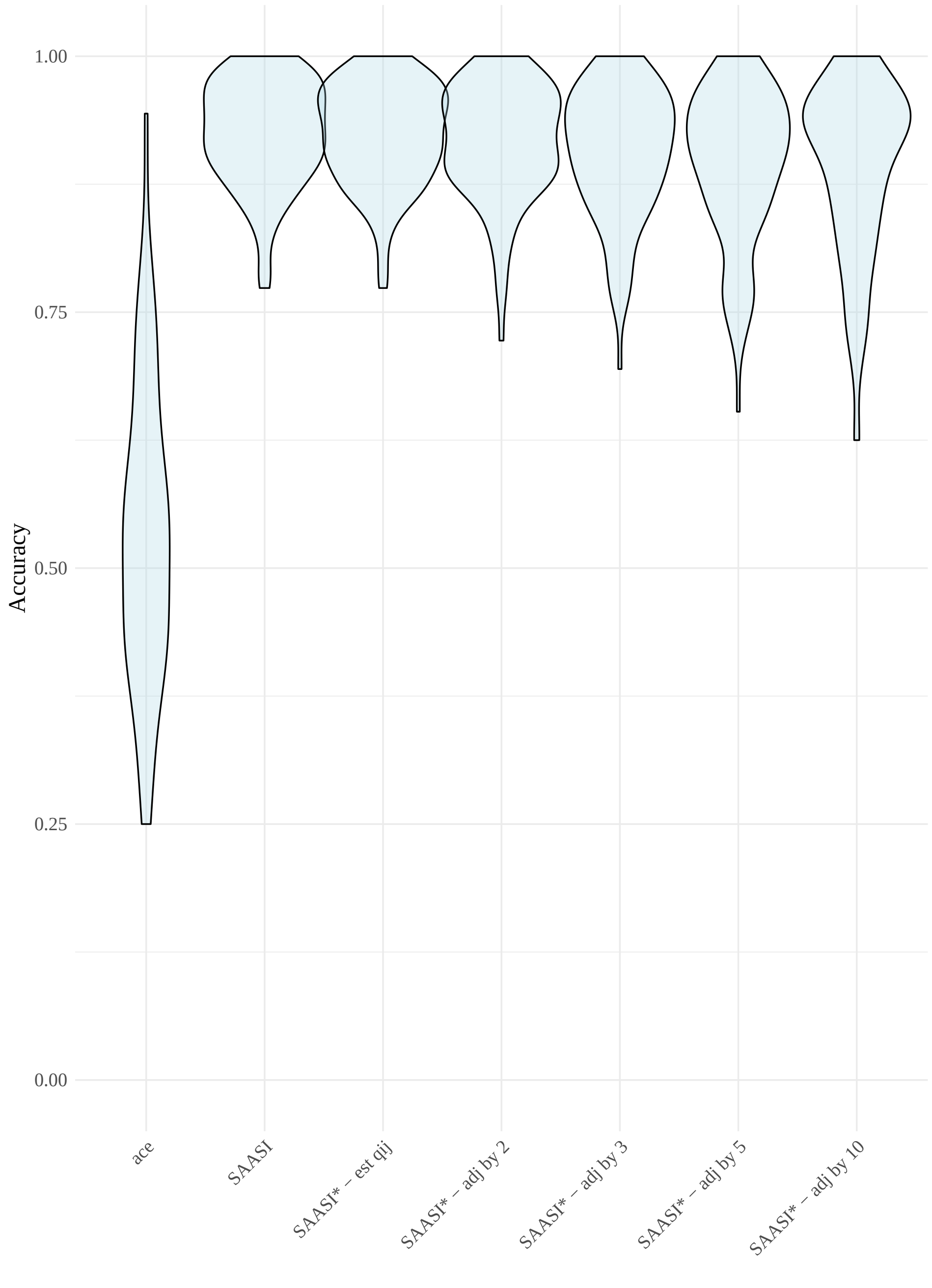


**Figure 3: Comparison of consensus accuracies for ancestral state inference using ace and SAASI under various transition rates adjustments.** state 1 is sampled 10 times less than the other states (three states in total). Simulations use $q_{ij} = 0.2, \forall i, j$. From left to right: ace; SAASI with true rates; SAASI* with estimated transition rates from 'ace' ($q_{ij}^{ace}$); SAASI with adjusted transition rates of state 1 by a factor of $c$, ranging from $c = \{2, 3, 5, 10\}$.

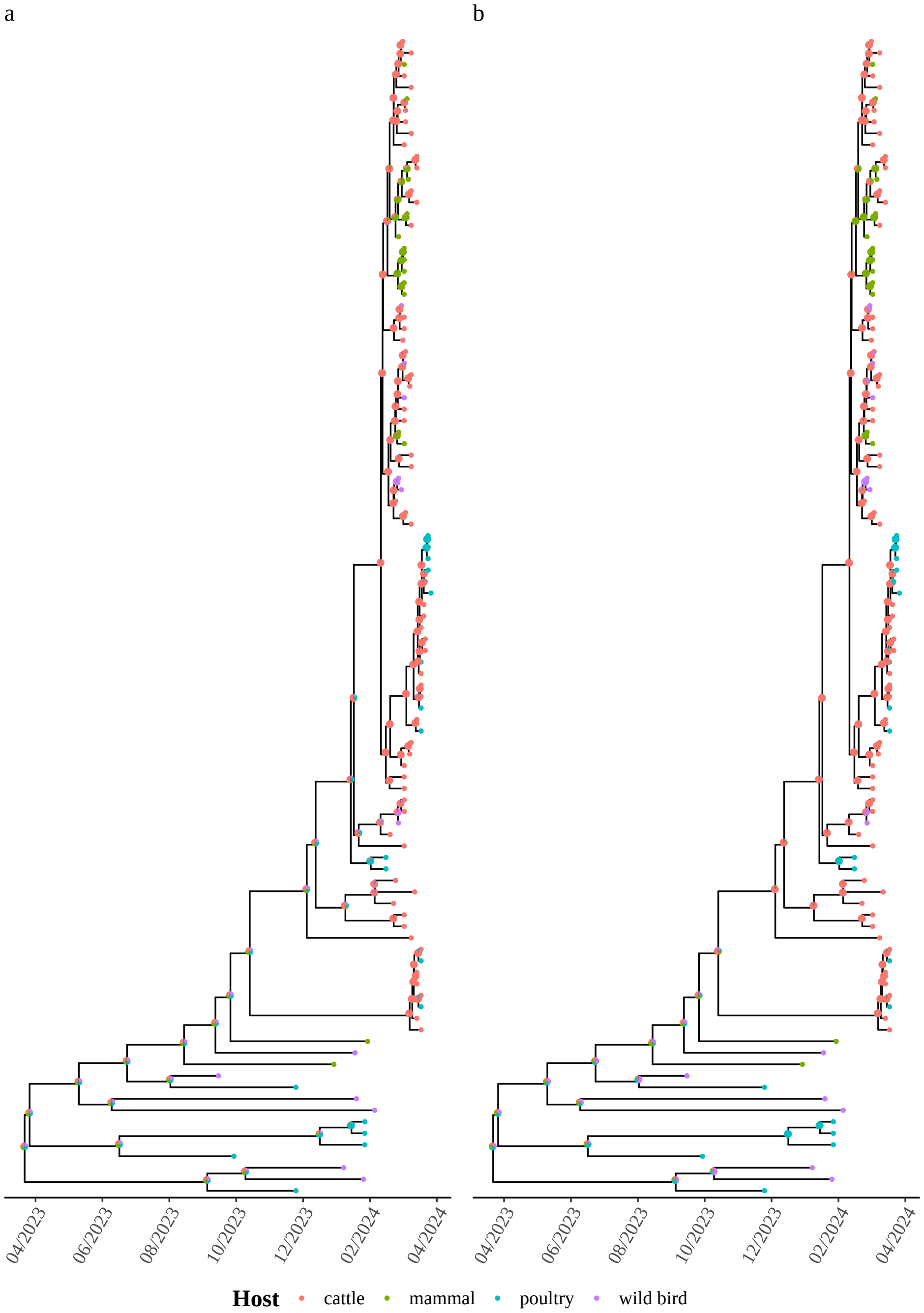


**Figure 4: Ancestral state inference of the H5N1 HA segment tree using ace and SAASI, assuming equal sampling across species.** a: ace; b: SAASI, equal sampling. Pie charts indicate the inferred probabilities of being in particular states.

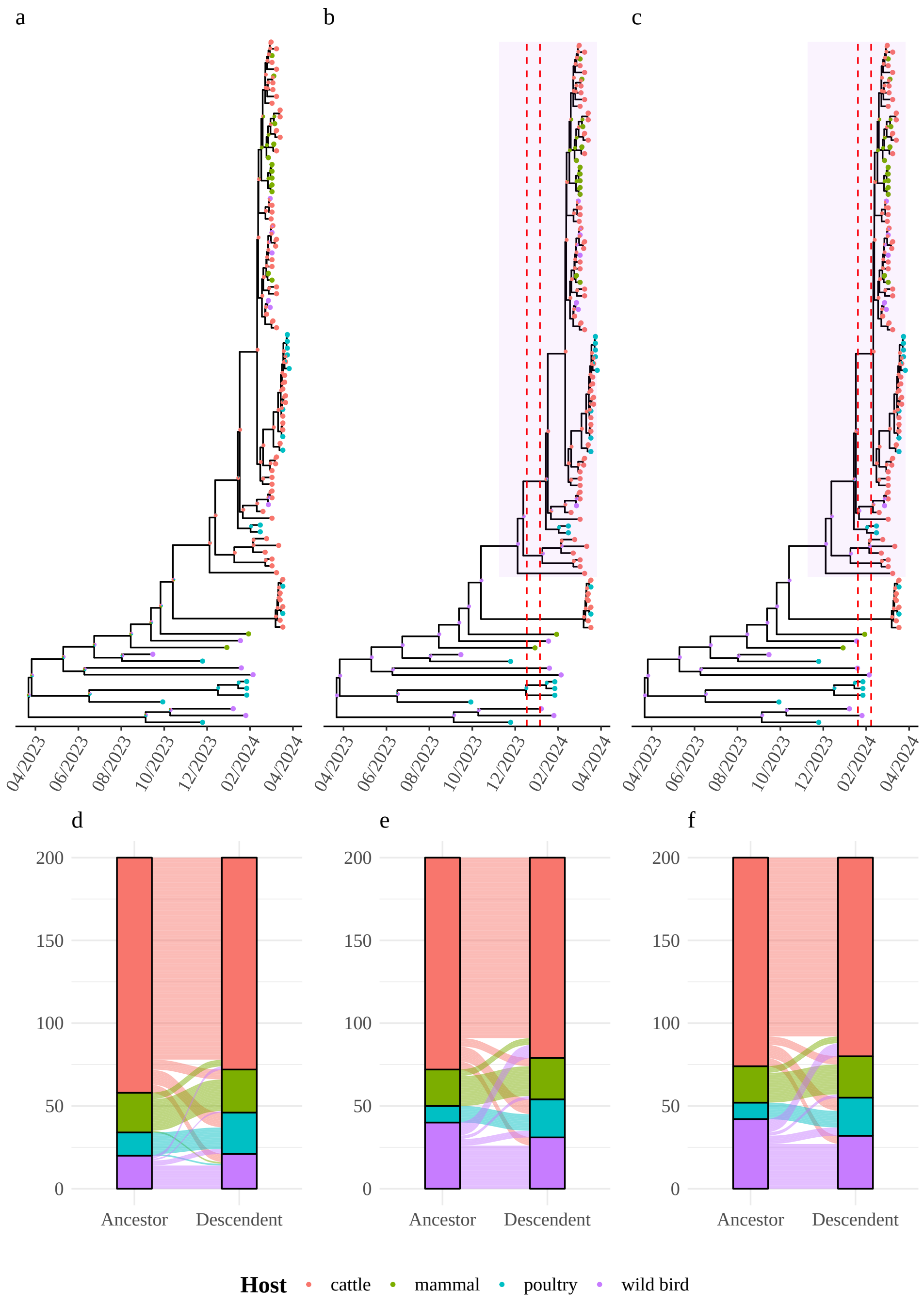


**Figure 5: Ancestral state inference of the H5N1 HA segment tree using SAASI under different species-level sampling models.** a: Inferred species hosts under equal sampling rates; b: Wild birds at one-tenth sampling; c: Wild birds at one-one hundredth sampling; d: Inferred viral transitions between host species in a; e: Inferred viral transitions between host species in b; f: Inferred viral transitions between host species in c. Pie charts indicate the inferred probabilities of being in particular states. Transition rates are equal between species. The dashed red lines indicate the key transition event from wild bird to cattle.

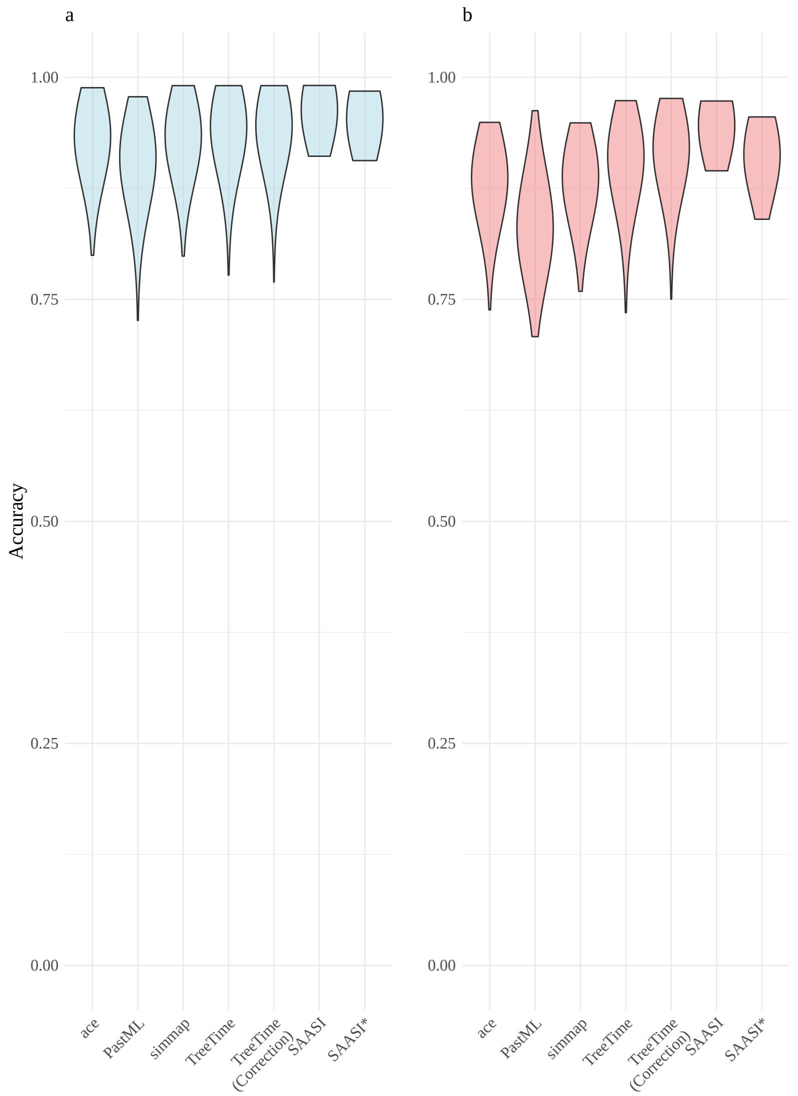


**Figure 6: Accuracy of ancestral state reconstruction methods under moderately biased sampling ($\psi_2 = 4\psi_1$) and state dependent diversification across 1000 simulated trees.** a: Consensus accuracy, defined as the fraction of correctly inferred ancestral node states. b: Probability accuracy, accounting for uncertainty in the node inference. Violin plots show the distribution of accuracy values across all simulations. ace and simmap use the equal-rates (ER) transition model. PastML employs the MPPA method with the F81 evolutionary model. TreeTime uses the mugration model with and without sampling bias correction (shown in parentheses). SAASI uses the true parameter values that generated the tree. SAASI* uses estimated speciation and extinction parameters but is provided with the true sampling rate. Trees were generated with speciation rates $\lambda_1 = 3$ and $\lambda_2 = 1.5$, extinction rates $\mu_1 = 0.1$ and $\mu_2 = 0.05$, transition rates $q_{12} = q_{21} = 0.3$, and sampling rates $\psi_1 = 0.25$ and $\psi_2 = 1.0$.

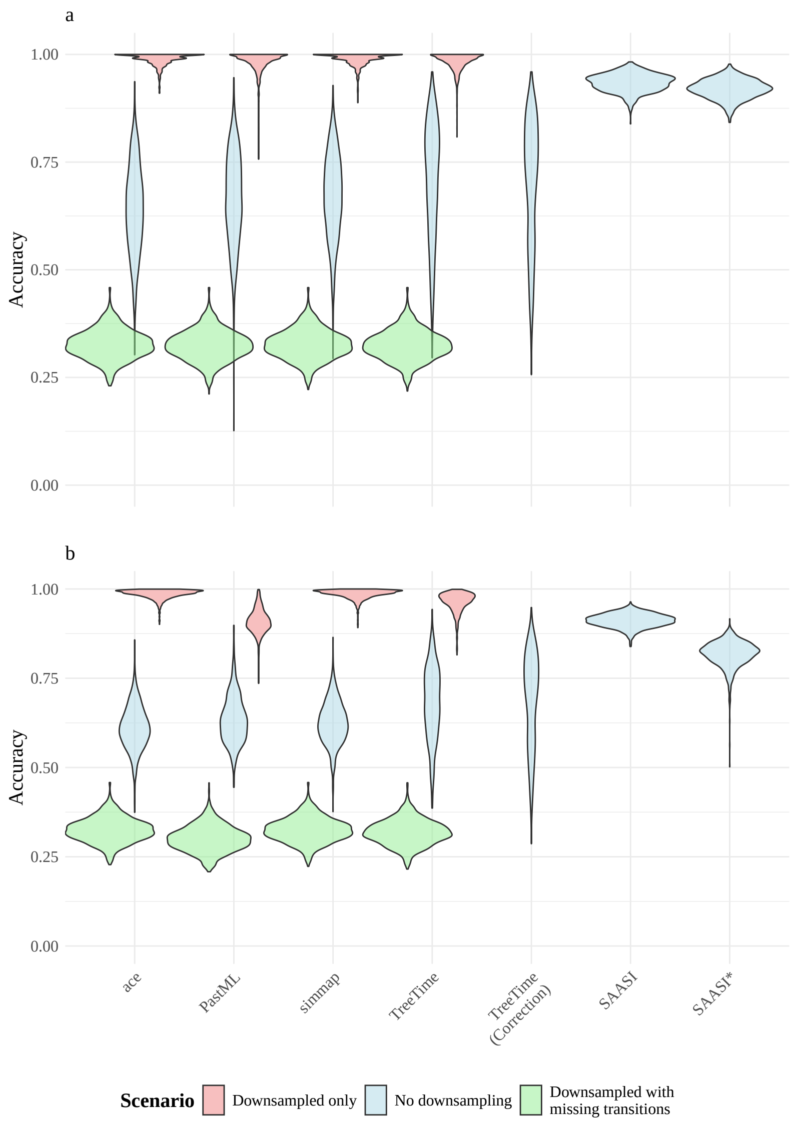


**Figure 7: Impact of downsampling on ancestral state reconstruction accuracy under highly biased sampling ($\psi_2 = 10\psi_1$) and state dependent diversification across 1000 simulated trees.** a: Consensus accuracy, defined as the fraction of correctly inferred ancestral node states. b: Probability accuracy, accounting for uncertainty in the node inference. Violin plots show the distribution of accuracy values across all simulations for three scenarios: (1) Downsampled only (red): accuracy calculated only on nodes present in the downsampled tree; (2) No downsampling (blue): accuracy on the original complete tree without any downsampling; (3) Downsampled with missing transitions (green): accuracy includes both downsampled nodes and transition events that occurred on branches removed during downsampling; . ace and simmap use the equal-rates (ER) transition model. PastML employs the MPPA method with the F81 evolutionary model. TreeTime uses the mugration model with and without sampling bias correction (shown in parentheses). SAASI uses the true parameter values that generated the tree. SAASI* uses estimated speciation and extinction parameters but is provided with the true sampling rate. Trees were generated with speciation rates $\lambda_1 = 3$ and $\lambda_2 = 1.5$, extinction rates $\mu_1 = 0.05$ and $\mu_2 = 0.1$, transition rates $q_{12} = q_{21} = 0.3$, and sampling rates $\psi_1 = 0.1$ and $\psi_2 = 1.0$.

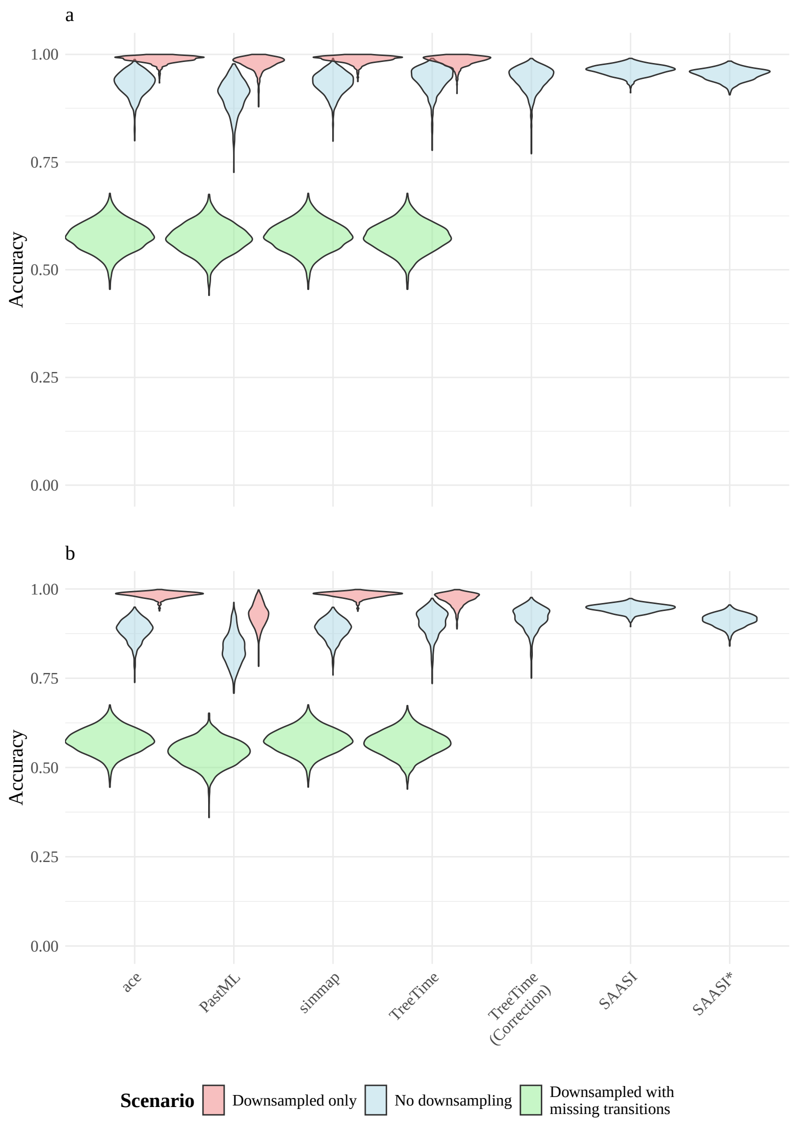


**Figure 8: Impact of downsampling on ancestral state reconstruction accuracy under moderate biased sampling ($\psi_2 = 4\psi_1$) and state dependent diversification across 1000 simulated trees.** a: Consensus accuracy, defined as the fraction of correctly inferred ancestral node states. b: Probability accuracy, accounting for uncertainty in the node inference. Violin plots show the distribution of accuracy values across all simulations for three scenarios: (1) Downsampled only (red): accuracy calculated only on nodes present in the downsampled tree; (2) Downsampled with missing transitions (green): accuracy includes both downsampled nodes and transition events that occurred on branches removed during downsampling; (3) No downsampling (blue): accuracy on the original complete tree without any downsampling. ace and simmap use the equal-rates (ER) transition model. PastML employs the MPPA method with the F81 evolutionary model. TreeTime uses the mugration model with and without sampling bias correction (shown in parentheses). SAASI uses the true parameter values that generated the tree. SAASI* uses estimated phylodynamic parameters but is provided with the true sampling rate. Trees were generated with speciation rates $\lambda_1 = 3$ and $\lambda_2 = 1.5$, extinction rates $\mu_1 = 0.05$ and $\mu_2 = 0.1$, transition rates $q_{12} = q_{21} = 0.3$, and sampling rates $\psi_1 = 0.25$ and $\psi_2 = 1.0$.

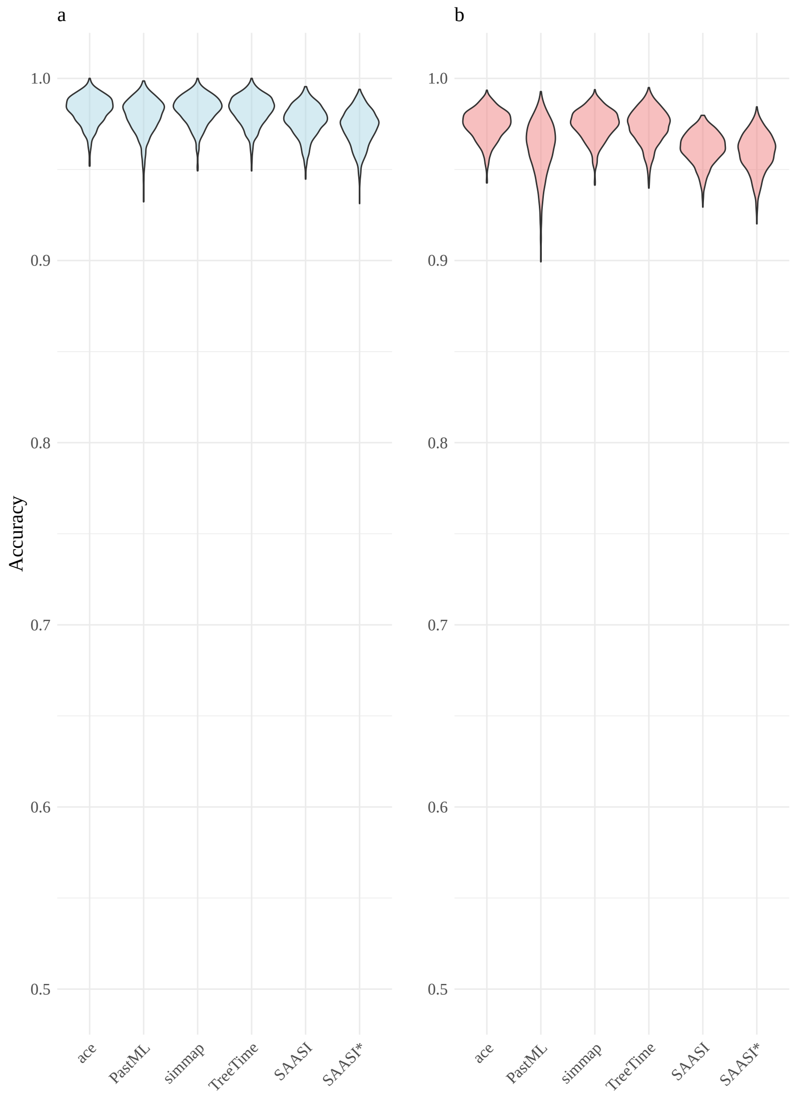


**Figure 9: Impact of downsampling on ancestral state reconstruction accuracy under unbiased sampling ($\psi_2 = \psi_1$) and state dependent diversification across 1000 simulated trees.** a: Consensus accuracy, defined as the fraction of correctly inferred ancestral node states. b: Probability accuracy, accounting for uncertainty in the node inference. Violin plots show the distribution of accuracy values across all simulations. ace and simmap use the equal-rates (ER) transition model. PastML employs the MPPA method with the F81 evolutionary model. TreeTime uses the mugration model with different sampling bias correction values (shown in parentheses). SAASI uses the true parameter values that generated the tree. SAASI* uses estimated speciation and extinction parameters but is provided with the true sampling rate. Trees were generated with speciation rates $\lambda_1 = 3$ and $\lambda_2 = 1.5$, extinction rates $\mu_1 = 0.05$ and $\mu_2 = 0.1$, transition rates $q_{12} = q_{21} = 0.3$, and sampling rates $\psi_1 = \psi_2 = 0.5$.

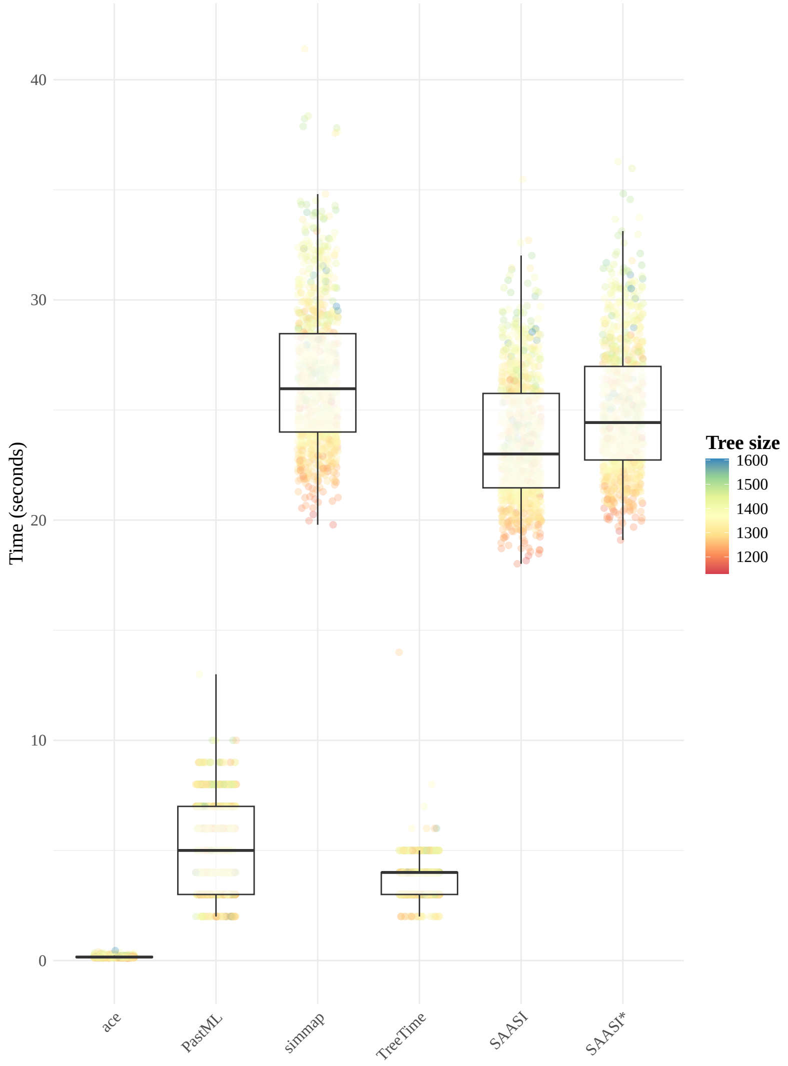


**Figure 10: Computational time for ancestral state reconstruction methods.** Running time is measured in seconds. Each point represents one simulation, with color indicating tree size (number of tips).

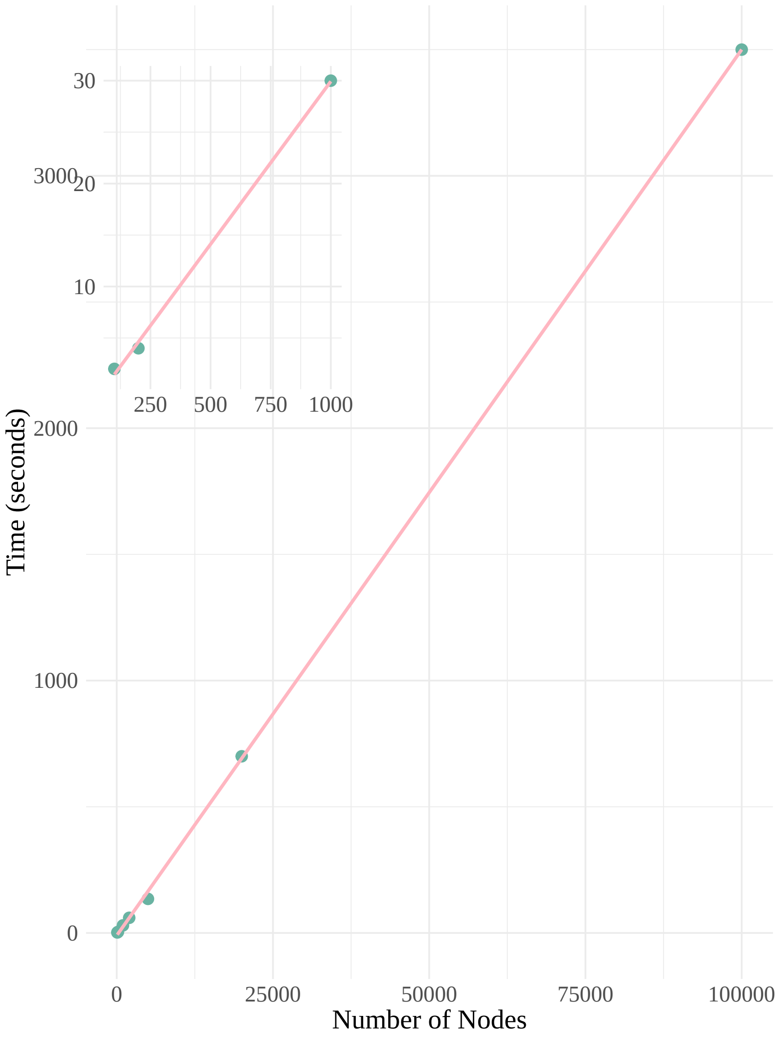


**Figure 11: Runtime of SAASI across trees of different sizes.** The x-axis represents the number of nodes, and the y-axis represents the times needed using SAASI. The red line shows the line of best fit. The small panel on the top left is a zoomed-in view of small tree sizes.

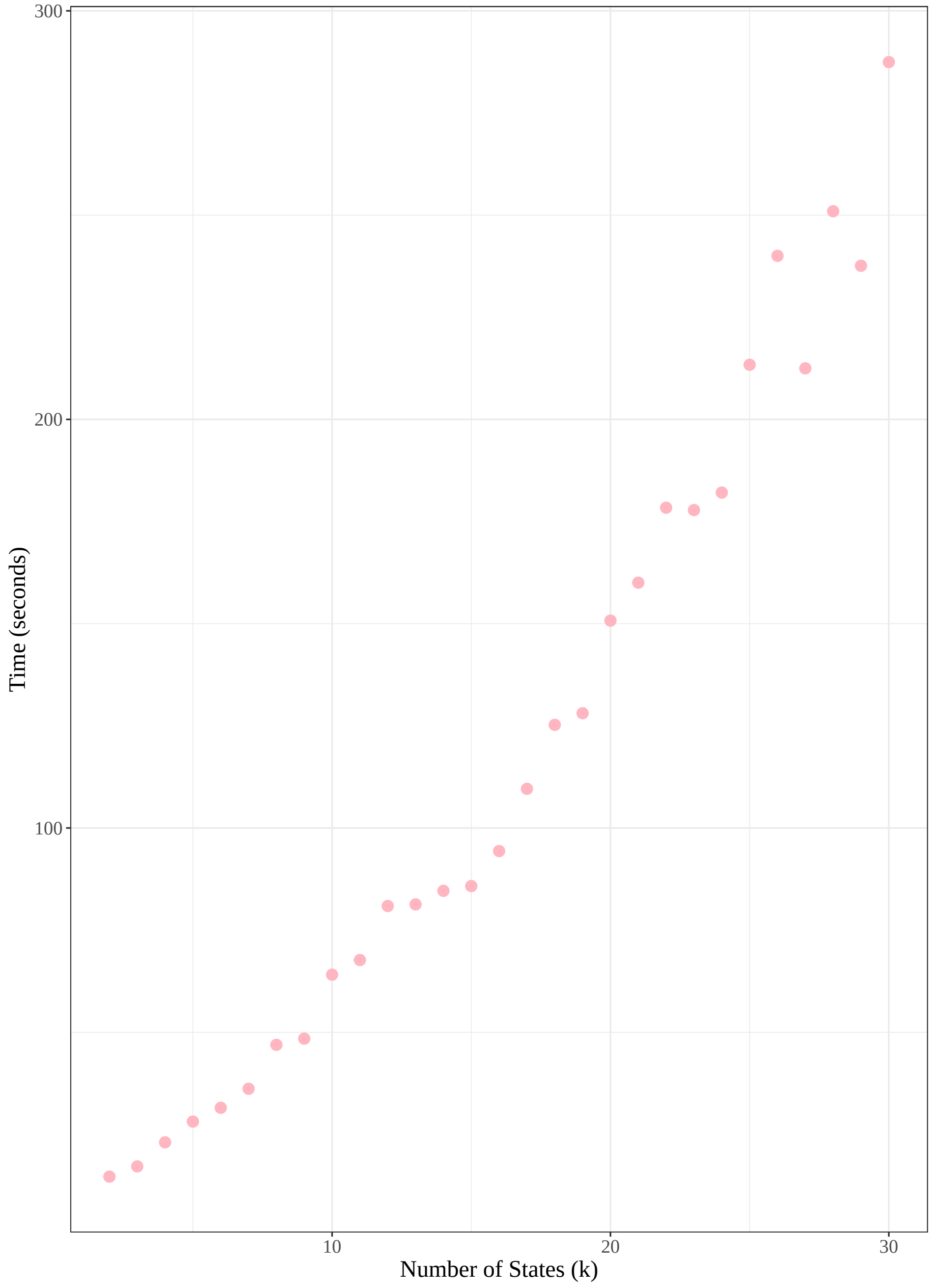


**Figure 12: Runtime of SAASI across trees of different numbers of states.** The x-axis represents the number of states, and the y-axis represents the times needed using SAASI.

| Accuracy | Consensuses Accuracy | | Probability Accuracy | |
|---|---|---|---|---|
| | Mean | SD | Mean | SD |
| ace | 0.641 | 0.103 | 0.611 | 0.061 |
| PastML | 0.664 | 0.108 | 0.634 | 0.067 |
| simmap | 0.670 | 0.098 | 0.626 | 0.060 |
| TreeTime | 0.707 | 0.134 | 0.678 | 0.102 |
| TreeTime (correction) | 0.699 | 0.147 | 0.689 | 0.130 |
| SAASI | 0.934 | 0.021 | 0.912 | 0.019 |
| SAASI* | 0.920 | 0.023 | 0.818 | 0.037 |

**Supplementary Table 1: Summary statistics of accuracy comparisons under high sampling bias, with a mean tree size of 463 tips.**

| Accuracy | Consensuses Accuracy | | Probability Accuracy | |
|---|---|---|---|---|
| | Mean | SD | Mean | SD |
| ace | 0.963 | 0.017 | 0.961 | 0.017 |
| PastML | 0.976 | 0.010 | 0.970 | 0.011 |
| simmap | 0.963 | 0.017 | 0.961 | 0.017 |
| TreeTime | 0.975 | 0.010 | 0.970 | 0.011 |
| TreeTime (correction) | 0.973 | 0.010 | 0.964 | 0.011 |
| SAASI | 0.976 | 0.007 | 0.964 | 0.008 |
| SAASI* | 0.975 | 0.008 | 0.968 | 0.010 |

**Supplementary Table 2: Summary statistics of accuracy comparisons under moderate sampling bias, with a mean tree size of 2669 tips.**

| | Subsampled tree only | | | | With subsampled tree | | | |
|---|---|---|---|---|---|---|---|---|
| Accuracy | Mean Cons. Acc. | SD Cons. Acc. | Mean Prob. Acc. | SD Prob. Acc. | Mean Cons. Acc. | SD Cons. Acc. | Mean Prob. Acc. | SD Prob. Acc. |
| ace | 0.990 | 0.014 | 0.985 | 0.013 | 0.327 | 0.034 | 0.325 | 0.034 |
| PastML | 0.983 | 0.025 | 0.910 | 0.034 | 0.325 | 0.035 | 0.301 | 0.036 |
| simmap | 0.989 | 0.014 | 0.985 | 0.014 | 0.327 | 0.034 | 0.325 | 0.035 |
| TreeTime | 0.984 | 0.021 | 0.963 | 0.028 | 0.325 | 0.034 | 0.318 | 0.035 |
| TreeTime (correction) | 0.699 | 0.147 | 0.689 | 0.130 | - | - | - | - |
| SAASI | 0.934 | 0.021 | 0.912 | 0.019 | - | - | - | - |
| SAASI* | 0.920 | 0.023 | 0.818 | 0.037 | - | - | - | - |

**Supplementary Table 3: Impact of downsampling on accuracy under high sampling bias: comparison between downsampled only, no downsampling, and downsampled with missing transitions datasets.** The accuracies calculated in TreeTime, SAASI, and SAASI* only consider the tree under no downsampling.

| | Subsampled tree only | | | | With subsampled tree | | | |
|---|---|---|---|---|---|---|---|---|
| Accuracy | Mean Cons. Acc. | SD Cons. Acc. | Mean Prob. Acc. | SD Prob. Acc. | Mean Cons. Acc. | SD Cons. Acc. | Mean Prob. Acc. | SD Prob. Acc. |
| ace | 0.988 | 0.009 | 0.984 | 0.008 | 0.578 | 0.033 | 0.575 | 0.033 |
| PastML | 0.980 | 0.015 | 0.927 | 0.030 | 0.572 | 0.034 | 0.542 | 0.035 |
| simmap | 0.988 | 0.009 | 0.984 | 0.008 | 0.578 | 0.033 | 0.575 | 0.033 |
| TreeTime | 0.985 | 0.012 | 0.972 | 0.017 | 0.576 | 0.033 | 0.568 | 0.034 |
| TreeTime (correction) | 0.973 | 0.010 | 0.964 | 0.011 | - | - | - | - |
| SAASI | 0.976 | 0.007 | 0.964 | 0.008 | - | - | - | - |
| SAASI* | 0.975 | 0.008 | 0.968 | 0.010 | - | - | - | - |

**Supplementary Table 4: Impact of downsampling on accuracy under moderate sampling bias: comparison between downsampled only, no downsampling, and downsampled with missing transitions datasets.** The accuracies calculated in TreeTime, SAASI, and SAASI* only consider the tree under no downsampling.

| Accuracy | Consensuses Accuracy | | Probability Accuracy | |
|---|---|---|---|---|
| | Mean | SD | Mean | SD |
| ace | 0.983 | 0.008 | 0.974 | 0.008 |
| PastML | 0.979 | 0.010 | 0.962 | 0.014 |
| simmap | 0.983 | 0.008 | 0.975 | 0.001 |
| TreeTime | 0.982 | 0.008 | 0.973 | 0.010 |
| SAASI | 0.977 | 0.008 | 0.962 | 0.009 |
| SAASI* | 0.973 | 0.010 | 0.959 | 0.011 |

**Supplementary Table 5: Summary statistics of accuracy comparisons under uniform sampling, with a mean tree size of 1360.**
