# Supplementary material for: SAASI: Sampling Aware Ancestral State Inference
Source: Nat Commun. 2026 May 11;17:6307. doi: 10.1038/s41467-026-72851-5 (PMC13376925; doi:10.1038/s41467-026-72851-5)
Supplement: Supplementary file 3 — Transparent Peer Review file [file 41467_2026_72851_MOESM3_ESM.pdf]

nature portfolio

Peer Review File

SAASI: Sampling Aware Ancestral State Inference

Corresponding Author: Mr Yexuan Song

This file contains all reviewer reports in order by version, followed by all author rebuttals in order by version.

Version 0:

Reviewer comments:

Reviewer #1

(Remarks to the Author)
Phylogenetic based reconstruction of ancestral epidemiological traits is an important tool to assess pathogen spread and host dynamics. However, such inferences have two important shortcomings: (1) the results of these analyses can be influenced by unbalanced sampling efforts and (2) many popular methods are too computationally intensive to scale with modern genome sequencing efforts. Song et al. have developed and tested a new method, Sampling Aware Ancestral State Inference (SAASI), that accounts for sampling bias in ancestral state inference and can scale with very large datasets. While SAASI is well conceived, further testing is needed to assess whether SAASI represents a significant improvement over established methods. My specific concerns with the manuscript are detailed below.

Major Comments

Simulation study.
Bayesian inference (with BEAST) and maximum likelihood inference with TreeTime (implemented in the Nextstrain analysis pipeline) are more commonly used for ancestral state inference of pathogen traits than the “ace” function in the ape R package. Unless the authors can provide strong justifications for the use of ace, the simulation studies would be more impactful if the performance of SAASI were compared to the TreeTime mugration model and phylogeographic diffusion in discrete space with BSSVS using BEAST.

The authors demonstrate that accuracy of SAASI is superior to ace when the sampling rates are known, but decreases as the specified sampling rate deviates from the true value. How likely is it that the sampling rate would be known, particularly from a large public dataset? If the sampling rate is known, a dataset could presumably be downsampled based on this rate. How does SAASI perform against other ancestral reconstruction methods when datasets are subset based on sampling rates (i.e. would the performance of ace be comparable to SAASI if the data is subset based on known sampling bias)?

Avian influenza.
The H5N1 outbreak in dairy cattle is an excellent choice for testing the effects of sampling imbalances on ancestral state inference. However, it is not fully clear how the results presented here compare to the BEAST analyses performed by Nguyen et al. (2025). Comparative results are briefly discussed in the methods section, but this should be moved to the discussion and expanded. For example, Nguyen et al. (2025) conclude that the H5N1 outbreak in dairy cattle is the result of a single spill over event from wild birds, whereas SAASI estimates 10-12 spillovers from wild birds to dairy cattle using the same data. These markedly different results should be discussed further.

Scalability to larger trees.
One of the most appealing features of SAASI is that it can be used with very large datasets. The authors do indeed present the runtimes of SAASI with various tree sizes, but without any comparisons to other methods. These comparisons are needed to assess whether SAASI is better suited to large datasets over other methods. Moreover, this study would benefit from benchmarking the performance of SAASI against other studies/methods that analysed large datasets (e.g. SARS-CoV-2) in a similar fashion to the influenza analyses.

Minor Comments
Figures 3, 4, S4 and S5: Size of the pie charts make it difficult to see the host transitions on the tree.
Lines 126-128: Was there a probability threshold to infer node states? Or is a state called with >0.50 probability?
Line 139: Revise to “…results are”

Lines 151-152: A single dashed line does not represent the date range November 2023-January 2024
Line 164: “0.52using ace”
Lines 447-448: Incomplete paragraph.

(Remarks on code availability)
The authors provide a very detailed tutorial for the installation and use of the SAASI R package here:
https://github.com/MAGPIE-SFU/saasi

Reviewer #2

(Remarks to the Author)
This study focuses on the application of the multitype-state speciation and extinction model with unequal sampling probabilities where the different states correspond to distinct host types or geographic demes. Simulations and the analysis of real data sets demonstrate the relevance of properly accounting for distinct sampling intensities across states.

This work deals with a highly topical subject, that of spatial sampling biases affecting statistical phylogeographic approaches. It also proposes a relevant probabilistic model that explicitly accommodates for variation in sampling intensities. The research performed in this study is of high quality and the manuscript reads really well. In particular, the description of the mathematics underlying the model of interest along with the corresponding post- and pre-order traversal algorithms is outstanding (although see minor point below). Yet, the model and the algorithms presented here are not new. In fact, they have already been thoroughly described and studied elsewhere (e.g., Freyman and Hohna, 2018 and very recently Vaughan and Stadler, 2025). More importantly, the lack of comparison between that approach and other models in statistical phylogeography, that were designed to tackle the very issue of sampling biases (De Maio et al., 2015 and Guindon and De Maio, 2021 in particular) is problematic in my opinion. Moreover, if I am not mistaken, the version of the model that is used in all experiments has the same rate of speciation and death for all states. This model is, at least conceptually, very similar to considering that the discrete trait (the deme or host type) evolves along the tree according to a continuous-time Markov chain with equilibrium frequencies set to the corresponding sampling frequencies in a BISSE-like model. All in all, there are obvious links between the approach proposed here and other models in phylogeography. These require clarification and a clear demonstration of the benefits of using one or the other approach.

Minor points

. Please provide a description of the differential equation for the extinction probability forward in time. This is a central (and not obvious, at least to me...) part of the pre-traversal algorithm and thus needs to be described in detail.

. Equation 7: Shouldn't the 2 in the numerator cancel out with that in the denominator?

. Also, the backward-in-time D terms in this equation seem to be evaluated at the start of edge e. Yet, the post-order traversal gives values of D at the end of each branch if I am not mistaken.

. \bar{\lambda}, introduced just before equation (7), is not defined.

Results from Fig 1.: do ace and simmap with equilibrium state frequencies set to the corresponding sampling frequencies give results similar to that of SAASI?

Labels of x-axis on Figure 2: replace "parmeters" with "parameters"

Scalability to larger trees: the authors rightfully mention that the running time of SAASI is expected to grow when including more states. Does the computing time of SASSI scale linearly, exponentially, or else with the number of demes? More generally, this section would benefit from the addition of results allowing to fully assess the performance of SAASI and other models when dealing with large data sets.

The 'ER' version of SAASI is used everywhere in this study. In case the different states correspond to demes, then using ER amounts to the Island Model whereby all pairs of demes (remote or close to each other) have the same rate of migration. Could the authors discuss what seems to be a limitation of the approach implemented here?

(Remarks on code availability)

Reviewer #3

(Remarks to the Author)
Ancestral reconstruction methods (ACR) are widely used in evolutionary biology, for phylogeography as described here, but also for many other questions, such as determining ancestral sequences or phenotypes. It is well known that sampling plays a very important role in these calculations and in the results. When sampling is highly heterogeneous, which is often the case, the standard solution with large datasets is to subsample overrepresented taxa. This article proposes a method for

directly integrating sampling rates into ancestral reconstruction, which is a significant advance, with a fast method that appears to give good results, both in simulations and on H5N1 data. I therefore recommend the publication of this article, but I believe that major changes are necessary to improve understanding of the proposed method and its limitations. It would also be good to go beyond phylogeography, which is just one application among many of ACR, and for which there are many models (e.g., https://doi.org/10.1016/j.ympev.2017.08.018).

Firstly, the results section should begin with a summary of the method and its innovations. Which model? How are the parameters estimated? What are the strategy and algorithmic complexity? Etc.? The method is based on approximations and shortcuts in relation to the chosen model, which must be presented here. This summary presentation is essential so that readers do not have to read the end of the article before starting to read the results. It is also important to summarize the characteristics of the simulated datasets and the reasons for these choices before giving the results.

In particular, the strategy of double post-order then pre-order tree traversal is very common in ACR (Downpass algorithm in MacClade, Maddison and Maddison; PastML, Ishikawa et al., MBE 2019). How does the strategy proposed here differ?

Some parts of the method are very formal (equations 1 to 10), while others on parameter estimation seem very approximate, based on a much simpler model than the initial SSE model. Under these conditions, it is difficult to get an idea of what the method and model actually are. If the sampling is uniform, what is the difference (theoretical, based on simulated data) with a standard method such as that implemented in ACE and PastML? With large trees, how does SASSI compare with these methods combined with subsampling?

In this respect, the simulations are all carried out without using the BiSSE model, with no difference between the rates for states 1 and 2. In this case, why add the complexity induced by the use of (Bi)SSE, if it is not taken into account? Can we use a simple birth-death model? Or, as is often the case in ACR, can we avoid using any tree model?

(Remarks on code availability)

Version 1:

Reviewer comments:

Reviewer #1

(Remarks to the Author)
The authors have done an excellent job revising their manuscript in accordance with the reviewers' suggestions. I only have two minor suggestions for the authors to consider before the manuscript is suitable for publications, which are listed below.

Line 125: “TreeTime, Treetime” edit for consistency.

Lines 259-272: It may be worth noting that ancestral reconstructions can be performed on a previously determined, fixed tree topology using Bayesian methods. However, this approach is still far more computationally expensive than ML methods.

(Remarks on code availability)
The authors provide detailed instructions to reproduce the results of their study.

Reviewer #2

(Remarks to the Author)
This revised version successfully addresses the comments made in the previous round of reviews in my opinion. I commend the authors once again for the quality of their work.

Below are a few minor remarks:

. Line 23: inference inference

. Fig. 2 is lacking labels on the y axis.

. Line 243: it is likely -> it is unlikely ?

. The finite difference equation preceding Equation 3 (p 14) needs to be amended. As is, it explicitly displays many terms in O(t^2), O(t^3) and O(t^4), which is at odd with the O(t^2) term at the end of it. Removing one of the other would solve the problem I guess.

. Line 477: simmap is run with stationary frequencies matching the sampling ratio. Correcting for unequal sampling frequencies would require instead setting the stationary frequencies to their true values (just like one uses the true sampling proportions for SAASI and SAASI*).

(Remarks on code availability)

Reviewer #4

(Remarks to the Author)
This is an interesting study, describing a novel approach to mitigate the impact of sampling bias on ancestral state inference, with applications to phylogeography. I am reviewing it in light of previous feedback provided by three other reviewers.

I concur that SAASI is statistically sound and a useful tool for future work (fig 3 nicely shows the use case, relative to other methods). I would be satisfied with how the authors have addressed comments from the reviewers and do not have major points myself. Instead I will raise a few points below that may or may not warrant minor edits:

- Label the y axis of fig 2 (this is essential). The measure of 'accuracy' as number of correctly inferred nodes was not obvious to me after reading the caption a few times.

- I disagree with Reviewer 2's point about comparisons with structured models. The structured coalescent (or birth-death) do account for sampling bias better, but the mechanism is fundamentally different in that they model population dynamic processes, and would be unsuitable for the use case of SAASI for large data sets.

- I also disagree that the comparison with Bayesian approaches (BSSVS etc.) is unfeasible. In practice, in maximum likelihood we constrain the tree, which can easily be done with Bayesian approaches (one could argue that then it is not fully Bayesian, but that is a different point). I would not insist that such comparison be done because the mechanism is not too different to stochastic mapping in ACE or other approaches. Moreover, BSSVS has many attractive properties, such as Bayesian regularisation, but its setup is not trivial (see Gao et al. 2023). I would be tempted to mention this in the manuscript.

References
Gao, J., May, M. R., Rannala, B., & Moore, B. R. (2023). Model misspecification misleads inference of the spatial dynamics of disease outbreaks. Proceedings of the National Academy of Sciences, 120(11), e2213913120.

(Remarks on code availability)
The repository is well documented and includes code in R and bash. I did not run it myself, but the documentation seems sufficient and functional links to original data on the readme file.

Open Access This Peer Review File is licensed under a Creative Commons Attribution 4.0 International License, which permits use, sharing, adaptation, distribution and reproduction in any medium or format, as long as you give appropriate credit to the original author(s) and the source, provide a link to the Creative Commons license, and indicate if changes were made.
In cases where reviewers are anonymous, credit should be given to 'Anonymous Referee' and the source.
The images or other third party material in this Peer Review File are included in the article's Creative Commons license, unless indicated otherwise in a credit line to the material. If material is not included in the article's Creative Commons

license and your intended use is not permitted by statutory regulation or exceeds the permitted use, you will need to obtain permission directly from the copyright holder.
To view a copy of this license, visit https://creativecommons.org/licenses/by/4.0/

Reviewer 1:

Phylogenetic based reconstruction of ancestral epidemiological traits is an important tool to assess pathogen spread and host dynamics. However, such inferences have two important shortcomings: (1) the results of these analyses can be influenced by unbalanced sampling efforts and (2) many popular methods are too computationally intensive to scale with modern genome sequencing efforts. Song et al. have developed and tested a new method, Sampling Aware Ancestral State Inference (SAASI), that accounts for sampling bias in ancestral state inference and can scale with very large datasets. While SAASI is well conceived, further testing is needed to assess whether SAASI represents a significant improvement over established methods. My specific concerns with the manuscript are detailed below.

Thank you. In line with your suggestions, and as detailed below, we have now included additional comparisons of SAASI to existing ASR methods, including procedures for adjusting for sampling bias. We welcome any further suggestions to facilitate comparison to existing work.

1. Simulation study.

Bayesian inference (with BEAST) and maximum likelihood inference with TreeTime (implemented in the Nextstrain analysis pipeline) are more commonly used for ancestral state inference of pathogen traits than the “ace” function in the ape R package. Unless the authors can provide strong justifications for the use of ace, the simulation studies would be more impactful if the performance of SAASI were compared to the TreeTime mugration model and phylogeographic diffusion in discrete space with BSSVS using BEAST.

We provide a more comprehensive comparison of SAASI with other maximum likelihood-based methods, including the TreeTime model, PastML, and simmap, as illustrated in Figure 2 and Supplementary Figures 6, 7, 8, and 9. We appreciate the suggestion to compare our methods with the Bayesian approach (e.g. BEAST). However, we have decided to restrict our comparisons to maximum likelihood (ML) methods. Bayesian and ML-based methods are suited for different situations, and ML methods are the only ones suitable for datasets large enough that Bayesian approaches are infeasible. Bayesian methods are suited for smaller datasets with phylogenetic uncertainty where sampling from a posterior distribution is the aim; this approach naturally brings in uncertainty in sampling fractions (and all other parameters), whereas the aim of SAASI is to account for known sampling differences. Furthermore, in a Bayesian posterior sample of phylogenetic trees (either with ancestral states or without), internal nodes are not comparable from one tree to the next, because the clade set

differs from tree to tree. There is therefore no way to compute an accuracy of the ancestral state reconstruction *per se* in the Bayesian methods (compared to a simulated true tree). Instead, comparisons would need to include whether the true clades were present in the posterior phylogeny samples *and* whether their state was correctly inferred (but without a reference to the "true" state for clades that do not occur in the true phylogeny). A direct comparison is therefore not strictly feasible, in addition to the use case difference.

2. The authors demonstrate that accuracy of SAASI is superior to ace when the sampling rates are known, but decreases as the specified sampling rate deviates from the true value. How likely is it that the sampling rate would be known, particularly from a large public dataset? If the sampling rate is known, a dataset could presumably be downsampled based on this rate. How does SAASI perform against other ancestral reconstruction methods when datasets are subset based on sampling rates (i.e. would the performance of ace be comparable to SAASI if the data is subset based on known sampling bias)?

We have included results (Supplementary Figures 7 and 8) showing the performance of each method after downsampling the tree when the sampling rates are known. We demonstrate that all methods perform well once the tree is downsampled to uniform sampling, provided that only the nodes preserved after downsampling are considered in the performance measure. If we instead take into account the lack of information about nodes that are dropped from the tree, then the "accuracy" drops significantly (i.e. the fraction of state-to-state transitions that can be uncovered is of course limited by the fraction of the nodes that remain after downsampling). Our findings indicate that even though downsampling the tree yields good accuracy *within the downsampled tree*, the overall accuracy decreases due to data loss.

We acknowledge that our method requires users to have prior knowledge about the sampling differences in their data, which is typically obtained at least qualitatively from surveillance data. If the sampling rates are unknown, a sensitivity analysis can be performed readily to assess the robustness of the results. As to how likely it is that sampling rates would be known, this is a good question. SAASI is motivated in part by the pandemic, where, while sampling rates would not be known perfectly, hospitalization data, deaths, testing policies, and some representative surveys such as the ONS survey in the UK meant that some information was available. In H5N1, while the sampling fractions are not known, it is certainly known that sampling is likely to be much lower in wild birds (as a fraction of infections) than in humans or domestic animal populations, and sensitivity analysis with SAASI is readily done. We find that the results are not sensitive to the precise under-sampling amount (x10, x20, etc) but that it matters greatly to consider lower sampling in wild birds at all.

3. Avian influenza.

The H5N1 outbreak in dairy cattle is an excellent choice for testing the effects of sampling imbalances on ancestral state inference. However, it is not fully clear how the results presented here compare to the BEAST analyses performed by Nguyen et al. (2025). Comparative results are briefly discussed in the methods section, but this should be moved to the discussion and expanded. For example, Nguyen et al. (2025) conclude that the H5N1 outbreak in dairy cattle is the result of a single spill over event from wild birds, whereas SAASI estimates 10-12 spillovers from wild birds to dairy cattle using the same data. These markedly different results should be discussed further.

We agree, and we have extended our discussion about the differences found between our analysis and that of Nguyen et al. (2025) (see lines 229-23). In analyzing this data our aim is to illustrate SAASI with a real-world example, where one species is likely to have had a substantially higher sampling rate (cattle) than another species (wild birds), though the sampling difference between wild birds and cattle remains uncertain. We do not undertake to re-analyze the full H5N1 dataset by Nguyen et al (which at the time of our work was available as a preprint), whose data were shared generously with the scientific community, and who should take precedence in publication of comprehensive analyses. We reached out to the authors of that group about SAASI and to let them know that our proof-of-principle SAASI analysis was forthcoming, and received positive replies. The data have already been re-analyzed by another group (see https://virological.org/t/preliminary-report-on-genomic-epidemiology-of-the-2024-h5n1-influenza-a-virus-outbreak-in-u-s-cattle-part-1-of-2/970 and the accompanying part 2).

Because we do not analyze the full dataset, we regard our H5N1 results as an illustration of SAASI and as indicative of the likely impact of accounting for sampling differences on phylogeographic or host reconstructions.

Scalability to larger trees.

One of the most appealing features of SAASI is that it can be used with very large datasets. The authors do indeed present the runtimes of SAASI with various tree sizes, but without any comparisons to other methods. These comparisons are needed to assess whether SAASI is better suited to large datasets over other methods. Moreover, this study would benefit from benchmarking the performance of SAASI against other studies/methods that analysed large datasets (e.g. SARS-CoV-2) in a similar fashion to the influenza analyses.

We have included a runtime comparison between each method (Supplementary Figure 10). We find that SAASI has a similar computation time to simmap, but has a

substantially higher running time than ace, PastML and TreeTime. However, at under a minute in these comparisons, it remains computationally feasible for large datasets (lines 155-173).

Minor Comments

4. Figures 3, 4, S4 and S5: Size of the pie charts make it difficult to see the host transitions on the tree.

We resize the pie charts to prevent overlap. See Figures 3, 4, Supplementary Figures 4 and 5.

5. Lines 126-128: Was there a probability threshold to infer node states? Or is a state called with >0.50 probability?

For the consensus probability, we choose the state with the highest probability (lines 497-499).

6. Line 139: Revise to "…results are"

We modify the sentence.

7. Lines 151-152: A single dashed line does not represent the date range November 2023-January 2024

We revise the figures to include two red dashed lines to represent a time range. See Figures 3 and Supplementary Figure 5.

8. Line 164: "0.52using ace"

We revise the sentence.

9. Lines 447-448: Incomplete paragraph.

We rearrange the paragraph to make it complete and clear (lines 555-558).

Reviewer #1 (Remarks on code availability):

The authors provide a very detailed tutorial for the installation and use of the SAASI R package here: https://github.com/MAGPIE-SFU/saasi

Reviewer 2:

This study focuses on the application of the multitype-state speciation and extinction model with unequal sampling probabilities where the different states correspond to distinct host types or geographic demes. Simulations and the analysis of real data sets demonstrate the relevance of properly accounting for distinct sampling intensities across states.

Thank you. In line with your suggestions, and as detailed below, we have now included additional comparisons of SAASI to existing ASR methods, including procedures for adjusting for sampling bias.

This work deals with a highly topical subject, that of spatial sampling biases affecting statistical phylogeographic approaches. It also proposes a relevant probabilistic model that explicitly accommodates for variation in sampling intensities. The research performed in this study is of high quality and the manuscript reads really well. In particular, the description of the mathematics underlying the model of interest along with the corresponding post- and pre-order traversal algorithms is outstanding (although see minor point below). 1. Yet, the model and the algorithms presented here are not new. In fact, they have already been thoroughly described and studied elsewhere (e.g., Freyman and Hohna, 2018 and very recently Vaughan and Stadler, 2025).

Pre- and post-order traversal algorithms are not new; numerous methods have been developed based on this idea. In our study, we identify that the pre-order traversal algorithm for the tilde{D} in speciation and extinction models is conditioned on the tree topology and should not contain events that alter the tree topology. We have clarified this contribution in the methods section and briefly in the discussion (eg lines 220-223). Our contribution is to show how the probability should be calculated rigorously. In Vaughan and Stadler, since they are sampling from the stated posterior distributions, they give the forms of those distributions, but do not give the derivation for allocating states to internal nodes.

2. More importantly, the lack of comparison between that approach and other models in statistical phylogeography, that were designed to tackle the very issue of sampling biases (De Maio et al., 2015 and Guindon and De Maio, 2021 in particular) is problematic in my opinion.

We appreciate the suggestion to compare our methods with the Bayesian approach (e.g. BASTA in BEAST). Please see the response above, which (to summarise) is that SAASI and Bayesian approaches are applicable to contrasting situations (both in dataset size

and in phylogenetic and sampling uncertainty), and produce results that are not directly comparable.

3. Moreover, if I am not mistaken, the version of the model that is used in all experiments has the same rate of speciation and death for all states. This model is, at least conceptually, very similar to considering that the discrete trait (the deme or host type) evolves along the tree according to a continuous-time Markov chain with equilibrium frequencies set to the corresponding sampling frequencies in a BISSE-like model.

Thank you for the suggestion. Our original intention is to illustrate the effect of biased sampling; hence, we set the other parameters equal. We have now rerun our simulations, considering different speciation and extinction rates for all states. See Figure 2, Supplementary Figures 6, 7, 8, and 9. A detailed description of the simulation parameters can be found in lines 446-475.

4. All in all, there are obvious links between the approach proposed here and other models in phylogeography. These require clarification and a clear demonstration of the benefits of using one or the other approach.

We add more inference methods and compare them to SAASI, including TreeTime, PastML and simmap (Figure 2 and Supplementary Figure 6). We include the results (Supplementary Figures S7 and S8) showing the performance of each method after downsampling the tree when the sampling rates are known. All methods perform well once the tree is downsampled to uniform sampling, provided that only the nodes preserved after downsampling are taken into account, as described above; naturally, other nodes are lost. We further conduct a simulation in which the states have equal sampling rates and find that all methods infer the internal nodes correctly (Supplementary Figure 9). To have a better understanding of the computation time of SAASI, we also compare it to other methods and find that SAASI has a similar running time to simmap (Supplementary Figure 10).

Minor points

5. Please provide a description of the differential equation for the extinction probability forward in time. This is a central (and not obvious, at least to me...) part of the pre-traversal algorithm and thus needs to be described in detail.

We describe the extinction probability in the pre-order algorithm (lines 394-395)

6. Equation 7: Shouldn't the 2 in the numerator cancel out with that in the denominator?

Thank you for catching this. Yes, the 2 in the numerator cancels out with that in the denominator (line 410).

7. Also, the backward-in-time D terms in this equation seem to be evaluated at the start of edge e. Yet, the post-order traversal gives values of D at the end of each branch if I am not mistaken.

Yes, the backward-in-time D is evaluated at the start of the edge e towards its parent node. We save the values and use them to initialize the pre-order traversal algorithm for each internal node (lines 411-414).

8. \bar{\lambda}, introduced just before equation (7), is not defined.

Thank you for pointing this out. We have changed \bar{\lambda} to the sum of all the speciation events that could occur (Line 408)

9. Results from Fig 1.: do ace and simmap with equilibrium state frequencies set to the corresponding sampling frequencies give results similar to that of SAASI?

We did not find a way to manually set the equilibrium state frequencies for ace other than modifying the source code. We now set the equilibrium state frequencies to the corresponding sampling frequencies for the simmap method (lines 477-478).

10. Labels of x-axis on Figure 2: replace "parmeters" with "parameters"

We have changed Figure 2.

11. Scalability to larger trees: the authors rightfully mention that the running time of SAASI is expected to grow when including more states. Does the computing time of SAASI scale linearly, exponentially, or else with the number of demes? More generally, this section would benefit from the addition of results allowing to fully assess the performance of SAASI and other models when dealing with large data sets.

We now include a running time comparison to the other methods. We show that SAASI has a similar running time to the stochastic character mapping (simmap) method. ace, simmap, PastML and TreeTime have a faster running time. We also include the tree size in colours to show the correlation between tree size and running time (See Supplementary Figure 10). We evaluate SAASI's computational performance across different numbers of states from 2 to 30 (see Supplementary Figure 12); results suggest that running time is quadratic in the number of states.

12. The 'ER' version of SAASI is used everywhere in this study. In case the different states correspond to demes, then using ER amounts to the Island Model whereby all pairs of demes (remote or close to each other) have the same rate of migration. Could the authors discuss what seems to be a limitation of the approach implemented here?

We use equal rate models in the simulation study because the transition rates in the simulation are set to be equal. In general, we can use different transition rates, such as symmetric rates. We set non-equal transition rates for H5N1 (species) analysis, and also test how transition rate adjustment could affect the accuracy (lines 435-441, Supplementary Figure 3).

Reviewer 3:

Ancestral reconstruction methods (ACR) are widely used in evolutionary biology, for phylogeography as described here, but also for many other questions, such as determining ancestral sequences or phenotypes. It is well known that sampling plays a very important role in these calculations and in the results. When sampling is highly heterogeneous, which is often the case, the standard solution with large datasets is to subsample overrepresented taxa. This article proposes a method for directly integrating sampling rates into ancestral reconstruction, which is a significant advance, with a fast method that appears to give good results, both in simulations and on H5N1 data. I therefore recommend the publication of this article, but

1. I believe that major changes are necessary to improve understanding of the proposed method and its limitations. It would also be good to go beyond phylogeography, which is just one application among many of ACR, and for which there are many models (e.g., https://doi.org/10.1016/j.ympev.2017.08.018).

We appreciate the positive comment and the suggestion. We have revised the introduction (lines 20-22) to clarify the range of uses of ASR. We have now also included additional comparisons of SAASI to existing ASR methods, including procedures for adjusting for sampling bias. We do go beyond literal phylo*geography* in our H5N1 application where the state is the host organism, not just the geographic location.

2. Firstly, the results section should begin with a summary of the method and its innovations. Which model? How are the parameters estimated? What are the strategy and algorithmic complexity? Etc.? The method is based on approximations and shortcuts in relation to the chosen model, which must be presented here. This summary presentation is essential so that readers do not have to read the end of the article before starting to read the results. It is also important to summarize the characteristics of the simulated datasets and the reasons for these choices before giving the results.

We have provided a description of the model and parameter estimation before discussing our results (lines 84-116). We further discuss computational time in lines 155-163.

3. In particular, the strategy of double post-order then pre-order tree traversal is very common in ACR (Downpass algorithm in MacClade, Maddison and Maddison; PastML, Ishikawa et al., MBE 2019). How does the strategy proposed here differ?

To make a more apparent distinction between our method and other methods, we provide a detailed description of the calculation of probabilities. We identify that in the speciation and extinction models, the pre-order traversal algorithm should not include extinction events, as it is conditioned on the tree's topology. We state this in lines 220 - 223. One contribution of this work is to clearly derive this probability with this conditioning.

4. Some parts of the method are very formal (equations 1 to 10), while others on parameter estimation seem very approximate, based on a much simpler model than the initial SSE model. Under these conditions, it is difficult to get an idea of what the method and model actually are. If the sampling is uniform, what is the difference (theoretical, based on simulated data) with a standard method such as that implemented in ACE and PastML? With large trees, how does SAASI compare with these methods combined with subsampling?

We have added detailed descriptions of how estimation procedures are performed. We provide a comprehensive comparison of SAASI with other maximum likelihood-based methods, including the TreeTime migration model, PastML, and simmap, as illustrated in Figure 2, Supplementary Figures 6, 7, and 8. We also conduct a simulation with unbiased sampling and find that all methods infer the internal nodes correctly (Supplementary Figure 9). Mathematically comparing phylogeographic models under various simplifying assumptions to determine their structural relatedness is interesting (and in fact a direction we are already considering), but it is beyond the scope of the current paper.

5. In this respect, the simulations are all carried out without using the BiSSE model, with no difference between the rates for states 1 and 2. In this case, why add the complexity induced by the use of (Bi)SSE, if it is not taken into account? Can we use a simple birth-death model? Or, as is often the case in ACR, can we avoid using any tree model?

Our original intention is to illustrate the effect of biased sampling, with other states being equal. We have now rerun our simulations by considering different speciation and extinction rates for all states. See Figure 2, Supplementary Figures 6, 7, and 8. A detailed description can be found in lines 446-475.

Reviewer’s Comments:

Reviewer #1 (Remarks to the Author)

The authors have done an excellent job revising their manuscript in accordance with the reviewers' suggestions. I only have two minor suggestions for the authors to consider before the manuscript is suitable for publications, which are listed below.

Thank you!

Line 125: “TreeTime, Treetime” edit for consistency.

Thank you for catching this. (Line 126)

Lines 259-272: It may be worth noting that ancestral reconstructions can be performed on a previously determined, fixed tree topology using Bayesian methods. However, this approach is still far more computationally expensive than ML methods.

Thank you for this helpful suggestion. We have clarified this in the Discussion. Specifically, we state that Bayesian phylogeographic methods can be applied on a fixed tree topology, but the analysis would still be very computationally expensive and the setup is nontrivial. (Lines 271-274)

(Remarks on code availability)

The authors provide detailed instructions to reproduce the results of their study.

Reviewer #2 (Remarks to the Author)

This revised version successfully addresses the comments made in the previous round of reviews in my opinion. I commend the authors once again for the quality of their work.

Thank you!

Below are a few minor remarks:

. Line 23: inference inference

Thank you for catching this. (Line 23)

. Fig. 2 is lacking labels on the y axis.

Thank you for catching this. We have added the labels on the y-axis for Fig.2, as well as Fig.S3, Fig.S6, Fig.S7, and Fig.S8.

. Line 243: it is likely -> it is unlikely ?

Thank you for catching this. Yes, this should be unlikely. (Line 241)

. The finite difference equation preceding Equation 3 (p 14) needs to be amended. As is, it explicitly displays many terms in O(t^2), O(t^3) and O(t^4), which is at odd with the O(t^2) term at the end of it. Removing one of the other would solve the problem I guess.

We have now removed O(t^2) at the end of the equation. (Line 357)

. Line 477: simmap is run with stationary frequencies matching the sampling ratio. Correcting for unequal sampling frequencies would require instead setting the stationary frequencies to their true values (just like one uses the true sampling proportions for SAASI and SAASI*).

We have clarified what we did in the text. Specifically, divide the number of observed tips in each state by the state's sampling rate, normalize these values so that they sum to one, and thus define the root priors for simmap. This is probably the best guess we could make for the true values – sampling-adjusted fractions.
In addition, we also clarified that we used the uniform prior for SAASI and SAASI* in the text. (Lines 480-482 and Line 486)

(Remarks on code availability)
Reviewer #4 (Remarks to the Author)

This is an interesting study, describing a novel approach to mitigate the impact of sampling bias on ancestral state inference, with applications to phylogeography. I am reviewing it in light of previous feedback provided by three other reviewers.

I concur that SAASI is statistically sound and a useful tool for future work (fig 3 nicely shows the use case, relative to other methods). I would be satisfied with how the authors have addressed comments from the reviewers and do not have major points myself. Instead I will raise a few points below that may or may not warrant minor edits:

- Label the y axis of fig 2 (this is essential). The measure of 'accuracy' as number of correctly inferred nodes was not obvious to me after reading the caption a few times.

Thank you for catching this. We have added the labels on the y-axis for Fig.2, as well as Fig.S3, Fig.S6, Fig.S7, and Fig.S8.

- I disagree with Reviewer 2's point about comparisons with structured models. The structured coalescent (or birth-death) do account for sampling bias better, but the mechanism is fundamentally different in that they model population dynamic processes, and would be unsuitable for the use case of SAASI for large data sets.

We agree that structured coalescent and structured birth-death models address a different class of problems. We have clarified this in the discussion to emphasize that these approaches have different focuses. (Lines 265-274)

- I also disagree that the comparison with Bayesian approaches (BSSVS etc.) is unfeasible. In practice, in maximum likelihood we constrain the tree, which can easily be done with Bayesian approaches (one could argue that then it is not fully Bayesian, but that is a different point). I would not insist that such comparison be done because the mechanism is not too different to stochastic mapping in ACE or other approaches. Moreover, BSSVS has many attractive properties, such as Bayesian regularisation, but its setup is not trivial (see Gao et al. 2023). I would be tempted to mention this in the manuscript.

References
Gao, J., May, M. R., Rannala, B., & Moore, B. R. (2023). Model misspecification misleads inference of the spatial dynamics of disease outbreaks. Proceedings of the National Academy of Sciences, 120(11), e2213913120.

Thank you for this helpful suggestion. We have made it clear in the Discussion. Specifically, we state that Bayesian phylogeographic methods can be applied on a fixed tree topology, but they are still more computationally demanding than maximum likelihood methods. (Lines 271-274)

(Remarks on code availability)

The repository is well documented and includes code in R and bash. I did not run it myself, but the documentation seems sufficient and functional links to original data on the readme file.
